# WEALTH, DEBT, AND COGNITIVE AND SELF-CARE DIFFICULTY AMONG AMERICANS AGED 65+

**DOI:** 10.1093/geroni/igae098.3767

**Published:** 2024-12-31

**Authors:** Connor Sheehan, Jiwon Jang, Joseph Saenz, Dylan Connor

**Affiliations:** Arizona State University, Scottsdale, Arizona, United States; Arizona State University, Tempe, Arizona, United States; Arizona State University, Phoenix, Arizona, United States; Arizona State University, Arizona State University, Arizona, United States

## Abstract

As the American population ages, the number of individuals with cognitive and self-care challenges is projected to increase substantially, stressing the importance of understanding their social determinants. Previous research has connected socioeconomic status, specifically educational attainment and income, to cognitive and self-care problems. However, considerably less research has examined the role of wealth in older adulthood. This is an oversight, as wealth reflects cumulative life course exposure and accumulation of socioeconomic resources, and debt may be a unique stressor. We use novel geographic machine learning-based estimates of wealth for every American household to predict cognitive and self-care difficulties among Americans aged 65+. We linked GEOWEALTH data, a machine learning estimate of household wealth, to the 2019-2021 American Community Survey (n=742,348) to create one of the largest data sources of wealth and health heretofore analyzed. Regression models predict cognitive and self-care difficulties adjusting for demographic and socioeconomic characteristics, including income and education. Cognitive and self-care difficulties decreased with increasing wealth, while being in debt nearly doubled the probability of these difficulties at every age. This pattern is amplified among White Americans and those in areas with concentrated debt. Wealth consistently shows stronger associations with cognitive and self-care difficulties than educational attainment or income. Our findings emphasize the importance of wealth and debt for well-being in older adulthood. Future research should explore the directionality of this association and the impact of different sources of wealth and debt.

